# Developing a Smartphone Application That Promotes Responsible Short-Acting Beta2-Agonist Use in People with Asthma: A Participatory Design

**DOI:** 10.3390/ijerph19148496

**Published:** 2022-07-12

**Authors:** Liselot N. van den Berg, Cynthia Hallensleben, Niels H. Chavannes, Anke Versluis

**Affiliations:** 1 Department of Public Health and Primary Care, Leiden University Medical Center, 2333 ZD Leiden, The Netherlands; c.hallensleben@lumc.nl (C.H.); n.h.chavannes@lumc.nl (N.H.C.); a.versluis@lumc.nl (A.V.); 2 National eHealth Living Lab (NeLL), 2333 ZD Leiden, The Netherlands

**Keywords:** asthma, primary care, SABA overuse, application, eHealth, participatory design, development process

## Abstract

Around 339 million people worldwide have asthma, and 50% have uncontrolled asthma. One trait of uncontrolled asthma, often seen in primary care, is short-acting β2-agonist (SABA) overuse, defined as using SABA more than twice a week. SABA overuse can cause adverse health effects. An application could help patients gain more insight into their SABA use. Engaging stakeholders during the development is important to maximize the usability of and adherence to an application. This study describes the development process of an application that promotes responsible SABA use in people with asthma, using a participatory design. Different stakeholder groups were involved in two iterative development cycles. In the first cycle, four end-users evaluated the app’s prototype. During the second cycle, five end-users were interviewed about the usability of the new version. Resulting in an app that allows patients to register SABA use, asthma symptoms, and symptom triggers. A graph shows how these factors are related, and end-users can show the graph to their physician to facilitate communication. Medication use is compared to the medical guidelines or, when applicable, to the advice given by the users’ healthcare professionals. End-users found the app helpful. Research into the usability and effectiveness of the app in a bigger sample will follow.

## 1. Introduction

Asthma is a chronic inflammatory disease of the respiratory tracts estimated to affect 339 million people worldwide [1]. For the past 50 years, the first step of medical treatment was to prescribe a short-acting β2-agonist (SABA) for rapid relief from an asthma attack. SABAs does not have an anti-inflammatory effect on the respiratory tracts. For this, patients can use controller medication such as inhaled corticosteroid (ICS) [2,3]. In 2019, the Global Initiative for Asthma [2] changed its treatment guidelines and included an as-needed low dose ICS-formoterol in the first treatment step. In the Netherlands, the Dutch College of General Practitioners [3] followed the Global Initiative for Asthma in 2020 and changed its guidelines accordingly. The option to select ICS-formoterol was added because asthma control in clinical (primary care) practice is often suboptimal [3,4,5]. Around 50% of patients have (partially) uncontrolled asthma [5,6,7]. One trait of uncontrolled asthma is SABA overuse, defined as using SABA more than twice a week [3].

The amount of used SABA canisters predicts the number of asthma exacerbations [8,9]. Stanford et al. [8] showed that using SABA more than twice per week for six months was related to a two-fold increased risk of an asthma exacerbation. In addition, they found that each additional SABA canister increased the risk by 14 to 18%. Overuse of SABA not only leads to a higher risk of asthma exacerbations but, over time, also damages the respiratory tracts [9,10]. Furthermore, studies have shown a relationship between asthma exacerbations and asthma-related hospitalization and visits to the emergency department [11,12].

A common reason for SABA overuse is that patients often use their SABA to achieve rapid relief from an asthma attack instead of taking their controller medication [13,14,15]. In the INSPIRE study [14], 90% of the participants stated that they were confident in managing their asthma but wanted rapid relief from their symptoms. Similar results were found in the REALISE study [16], where more than 80% of the participants considered their asthma to be controlled, whereas around 40% of the participants had used their SABA more than three times in the previous week. Using SABA more than the recommended frequency of twice per week [2,3] may indicate a reliance on SABA [16]. Patients often learn from their healthcare professional (HCP) that they can use SABA for quick relief [13]. Since SABA is still one of the two options in the first treatment step [2,3], which could give patients the perception that SABA is the main treatment for their asthma [13]. As a result, a lot of SABA overuse is seen in primary care [17,18]. In patients who are in the process of an asthma diagnosis or just had their diagnosis, the HCP commonly chooses to start only SABA treatment instead of recommending an as-needed low dose ICS-formoterol [19,20]. This is also the case for patients who had their diagnosis for a longer period. Forty-four percent of the SABA over-users did not attend an asthma review in primary care last year [17]. Changing asthma medication is not achievable when an asthma review is not attended. All these factors might explain why overall adherence to ICS is around 50% and patients still rely on SABA [21]. Other reasons for SABA overuse are a lack of knowledge about asthma medication in general and a lack of insight into the actual frequency of medication use [13,22].

A personalized self-management application (app) could help patients gain more insight into their medication use [23,24]. Such a self-management tool could support patients to manage their disease daily [25,26] and can include education, self-monitoring, and (personalized) feedback [25,26,27,28]. Self-management tools and interventions have previously successfully been used in individuals with a chronic disease [29,30]. A systematic review found that mobile technology interventions have successfully improved adherence and clinical outcomes in asthma [31]. Furthermore, other studies show that self-management apps help to reduce the frequency of SABA use, increase SABA-free days, and improve asthma control [23,24]. Moreover, these tools can increase individuals’ confidence to manage asthma and improve their quality of life [25,27,32]. A smartphone application that helps patients get more insight into their SABA use while simultaneously promoting responsible SABA use may help to reduce SABA overuse. The objective of this study was to develop an app that helps asthma patients to get more insight into their SABA use and over time to help to decrease SABA overuse. This paper provides an overview of the development process of the app.

Although health apps are frequently downloaded [33], studies show that the actual use is suboptimal. Krebs and Duncan [33] showed that health-related apps had been downloaded by 58% of their study population; however, almost half (46%) no longer use these apps once downloaded. The main reasons are that it took too much time to enter data, loss of interest, and hidden costs. A systematic review [34] further showed that the attrition rates in self-management apps range from 9 to 26%. Frequent reasons for dropout are issues with technology (i.e., experiencing difficulties using the telephone or encountering application failures) and the burden of the input frequency (e.g., unwillingness to continue regular measurements) [34]. To stimulate optimal use of an app, it needs to fit into the daily life of the user [35]; however, there is often a discrepancy between the intended and actual app usability [35,36,37]. One of the reasons for this is that end-users are often not involved in the development process [35]. Due to this top-down development, problems in the app can come up after the launch [38]. For example, the inability of patients to find the information they are looking for, insufficient information, and language that does not suit their needs (i.e., a semantic mismatch between the app and the users) [39]. Developers can choose from various mobile app development models. A systematic review identified that state-based models, such as Waterfall, and agile methods are the most commonly used [40]. These traditional developing models include requirement activities, design, implementation, testing, deployment, and maintenance [40]. User input, however, is not included throughout the whole app development. Not sufficiently including end-users in the app development can lower usability and adherence [35]. Furthermore, people with limited (digital or health) literacy may not have the required degree of literacy to use a self-management app, which may worsen health inequalities [41]. Thus, engaging low-literate users during app development would be optimal to help decrease health inequalities. It is also essential to include other relevant stakeholders in the app development process, such as HCPs, as their needs may differ from the end-user needs [41].

Therefore, when developing health apps, end-users and other relevant stakeholders should be involved in the development process to improve the usability of and adherence to apps [35,42,43]. One way to accomplish increased usability and adherence is by using a participatory design [44]. In a participatory design process, technologies, such as apps, are developed closely with the relevant stakeholders [43,44,45]. Including different stakeholders, such as end-users, researchers, developers, and HCPs, boost the diversity of knowledge, experience, and value, positively impacting the development and design process [46]. A participatory design is a step-by-step process including planning, developing, testing, and giving feedback. It minimally uses two iterative cycles [43,44]. In each cycle, stakeholders are involved in testing and giving feedback. This study describes the development process of an application that promotes responsible SABA use in people with asthma, using a participatory design.

## 2. Materials and Methods

### 2.1. Study Design and Participants

This study had a qualitative descriptive design. Two iterative testing and feedback cycles were incorporated into the development process and are further described under the “app development process”. Individuals could participate if they (a) were 18 years or older and (b) had asthma. There were no further exclusion criteria. The study did not fall within the scope of the Dutch Medical Research Involving Human Subjects Act according to the Medical Ethics Committee of Leiden University Medical Centre (G21.068), because participants were not subjected to procedures, nor were they required to follow rules of behavior.

### 2.2. Procedure

A convenience sample of asthma patients was recruited by a research nurse (author CH) via the closed Facebook group *Asthma and Peers* in the Netherlands. A message was posted asking if people were interested in participating in a qualitative test session (i.e., first iterative cycle) to give feedback on an asthma app under development. Interested individuals were instructed to contact the research nurse via a personal Facebook message. Via email, they received information about the research. Three qualitative test sessions of 60 min were organized (i.e., two sessions with one participant and one session with two participants). Participants could try out the app prototype in the session while thinking aloud and answering questions. The participants received a gift card of 25 euros, and travel costs were reimbursed. The app prototype was also tested in individuals with low (digital) health literacy to ensure inclusiveness. This was done by Pharos, an institute with expertise in health disparities.

Four months after the first cycle, the second iterative cycle started. Individuals who participated in the first cycle and people who had been interested in participating in the first cycle but had been unable to attend were invited. Interested individuals were able to test the new version of the app in their daily life for a couple of days over a time span of one week. In-depth, semi-structured, individual interviews were held to get insight into the participants’ experiences with the app. Interviews were conducted via telephone and lasted between 30 and 45 min. Participants could indicate whether they wanted to be notified when the app was launched. No reimbursement was given. Informed consent from the participants to use their data was obtained.

### 2.3. App Development Process

**Figure 1:**
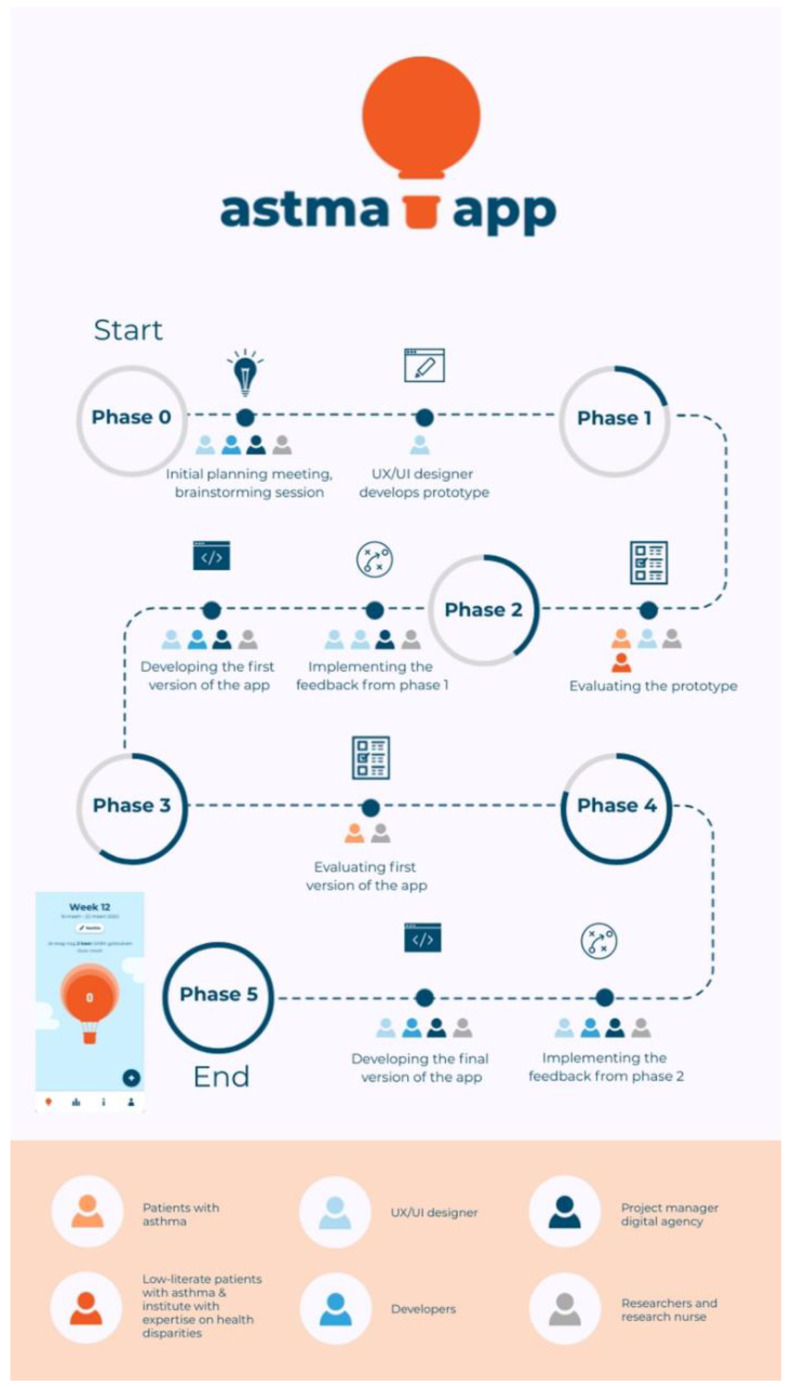
Overview of all the development phases of the Asthma app. Phases 0–1: first iterative cycle. Phases 2–5: second iterative cycle.

Figure 1 gives an overview of all the development phases, the subtasks per phase, and the stakeholders involved per subtask. Phases 0–1 formed the first iterative cycle, and phases 2–5 constituted the second iterative cycle.

### 2.4. Phase 0—Identifying the Problem and Developing the Prototype

**Figure 2:**
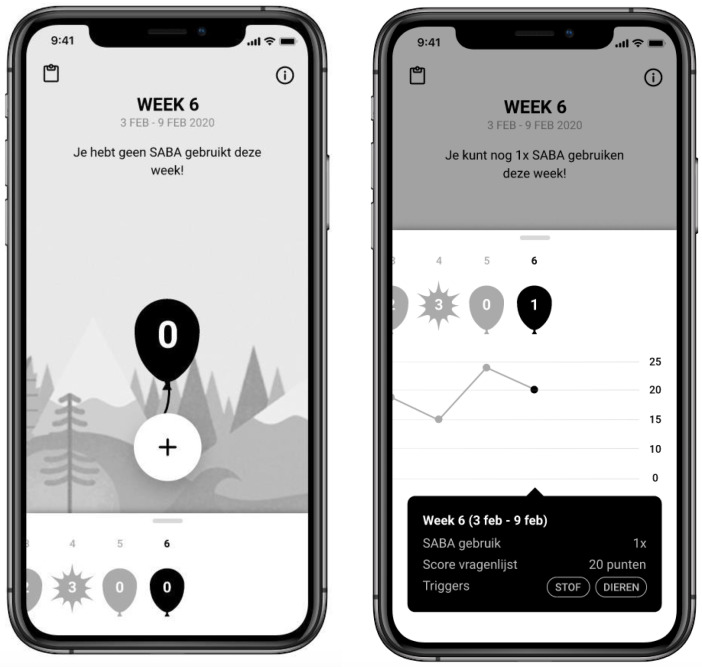
Prototype of the app.

The Global Initiative for Asthma announced the new treatment guidelines for asthma in 2019. The problem of SABA overuse and the underlying causes were further specified by a researcher (author AV) and a research nurse (author CH) via literature research. Next, a brainstorming session was held with developers and a UX/UI designer from a digital agency to discuss the new eHealth application and what functionalities were needed to promote responsible SABA use. The group established the following functionalities to be implemented in the app: users should be able to register their SABA use, they should be able to add symptoms and triggers when registering SABA use, and users should be made aware of the amount of SABA they can use per week, and they should be able to get a weekly overview of their SABA use and asthma symptoms. Based on this, the UX/UI designer developed a prototype with a landing page where users could register SABA and a page with a graphical overview (see Figure 2). On the landing page, the balloon showed how much SABA the user had taken that week. After every SABA registration, the balloon would inflate a little. The balloon would burst when the maximum dose of two SABAs per week, which is the standard recommendation [3], was exceeded. Additionally, textual information was shown, for example, “you have not yet used any SABA this week”. The other page consisted of (a) a graphical overview of the amount of asthma symptoms (assessed using the Control of Allergic Rhinitis and Asthma Test [CARAT]) [47,48], (b) the amount of SABA used per week, and (c) symptom triggers that were entered.

### 2.5. Phase 1.1—Evaluating the Prototype (Qualitative Test Sessions)

The purpose of the qualitative test sessions was to evaluate the prototype of the app. The three sessions were led by the UX/UI designer. Notes were taken by one of the researchers (author LvdB). First, the participants received a short introduction to the prototype. Second, they received a tablet with the app prototype and were asked to perform tasks to check the app functionality, such as “add one SABA inhalation” and “what was the score of the questionnaire in the fourth week?”. Additional questions were asked to get insight into the participants’ experience with the prototype (e.g., “how did you perceive the specific components of the app?” and “what did you miss?”).

### 2.6. Phase 1.2—Evaluating the Prototype (Institute with Expertise on Health Disparities)

In the session led by Pharos, two low-literate patients with asthma, who used medication, were shown the prototype. A similar set-up was used as in phase 1.1.

### 2.7. Phase 2—Implementing Feedback from Phase 1

After the first iterative cycle, the development team (i.e., the UX/UI designer, developers, project manager, researchers, and research nurse; see Figure 1) decided which feedback could be implemented in the app. To ensure that the most critical feedback was incorporated, the group identified how often a particular feedback point was given and whether it was feasible to address the feedback. The aim was to incorporate the most frequently given feedback, although addressing the feedback also needed to be feasible. For example, almost all participants wanted to register all their medication, not just asthma-related medication. This felt outside the project’s scope, which was to create an app to promote responsible SABA use, so this feedback was not implemented. The feasible feedback was implemented in the first version of the app by the development team.

### 2.8. Phase 3—Evaluating the First Version of the App

The first version of the app became available for participants a week before the in-depth interviews were held. The aim of the interviews was to gather information about the app’s usability. Questions were asked such as “what did you like about the app and which aspects did not work for you?” and “were all texts comprehensible?”.

### 2.9. Phase 4—Implementing Feedback from Phase 2

The same strategy as in phase 2 was used to prioritize the feedback. The sponsor of the app, a pharmaceutical company, also gave feedback about the first test version. However, they played no further role in the development of the app (see Funding statement for details). The feedback from the participants and the sponsor were taken into consideration by the development team. Afterward, the feasible feedback was implemented by the development team.

### 2.10. Phase 5—Final Version of the App

**Figure 3:**
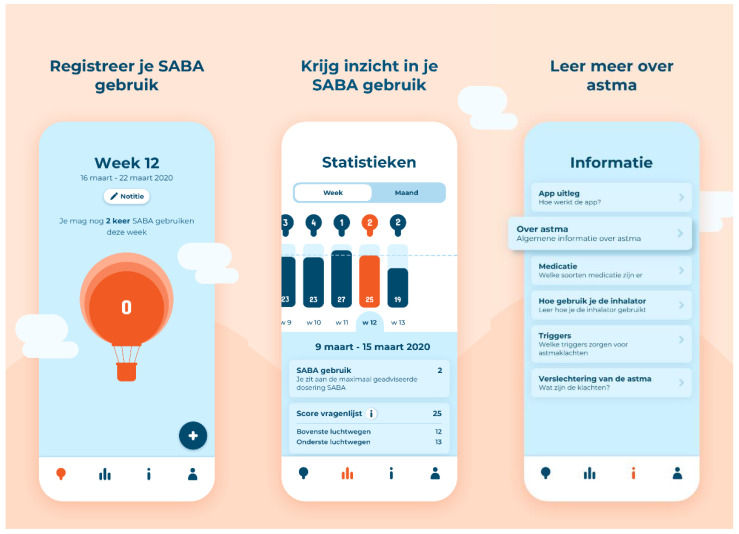
Visuals of the final version of the Asthma app. “Registreer je SABA gebruik” means “Register your SABA use”, “Krijg inzicht in je SABA gebruik” means “Get insight into your SABA use” and “Leer meer over astma” means “Learn more about asthma”.

The final version of the app is shown in Figure 3. The app allows users to register their SABA use directly. Moreover, asthma symptoms can be measured weekly, and participants can register the triggers of these symptoms at any time. A graphical overview shows how SABA use, asthma symptoms, and their triggers are related. In the app, the amount of SABA use is compared to the existing guidelines or, when applicable, to the advice given by an HCP. The guidelines advise using SABA twice per week [3]. Psychoeducation is also included in the app, covering topics such as “what is asthma”, “types of medication and their function”, and “asthma control”. The researchers created this written information on communication level 1B (on the scales of the Common European Framework of Reference for Languages [49]).

## 3. Results

In total, six female patients and two low-literate (gender unknown) patients with asthma co-created the asthma app using a participatory design. Three of the six female participants were involved in both the qualitative test session and the interview, two of them only participated in the interviews, and one female participant only participated in the qualitative test session. These results are discussed per iterative cycle. The input from the two low-literate patients is discussed separately under the “Pharos session”.

### 3.1. First Iterative Cycle

All four participants were interested in the app’s potential to track their medication use, symptoms, and triggers. They stated the app could be used to remind themselves about the amount of SABA they already used and to facilitate communication with the general practitioner and pulmonologist. Furthermore, the participants indicated that they currently do not have an effective tool to register their SABA use or an overview of medication use, symptoms, and triggers. Other strategies that participants used to register their medication were: making notes on their phone to know when the inhaler would be empty (*n* = 1), sometimes writing it down in a notebook (*n* = 1), and keeping track of medication using a pillbox (*n* = 1; other medication than an inhaler). Two of the participants stated that they did not know what they would name the app.

All remarks about the app prototype can be found in Table 1. The remarks that were not applicable (i.e., the development team would not be doing anything with the remark due to it (a) already being incorporated in the app or (b) not being applicable for the development of the app (e.g., “I always play with new apps first to see how they work”)) can be found in Appendix A, Table A1.

Participants were asked to register their SABA use. For all participants, it was clear how they could add a SABA dose to the prototype. Participants mentioned that they would immediately register their SABA use in the app because otherwise, they would forget. Another frequently made remark was that the amount of SABA advised is personal (“I need to use SABA more than once a day already”), which the prototype did not reflect. The development team (see Figure 1) found the second remark feasible (+; see Table 1), and in the next version of the app, users could personalize the maximum amount of SABA per week.

Participants received the question to share their thoughts about the balloon. Most participants (three out of four) found the warning about taking too much SABA appropriate and were not deterred by the bursting balloon. One participant found the balloon’s burst stigmatizing (see Table 1). The same participant also mentioned that a bigger balloon is not equal to less air. Based on these feedback points, the balloon was changed into a hot air balloon that gets smaller and descends to the bottom of the screen with every SABA registered.

All participants confirmed that the triggers in the graphical overview were clear to them. The participants mentioned that relevant triggers were grass pollen and trees (75%), the farm (25%), certain food in combination with hay fever (25%), cold (25%), and sneezing (25%), and exertion (25%). To keep the triggers manageable, the development team decided to use the same triggers used by the Lung Foundation Netherlands [50]. Furthermore, exertion was added as a trigger because up to 80% of people with asthma without anti-inflammatory treatment may have an exacerbation due to exercise [51].

In addition to the weekly overview, participants mentioned that they wanted to view the data in the graph per half-year, monthly, or per season. The development team decided to keep the app simple and intuitive for all users and chose one of the three suggested overviews. Monthly seemed the most logical because users could get lost in the high amount of data in a six-month or seasonal overview.

Participants were asked about the questionnaire to fill in asthma symptoms (this function was not yet incorporated in the prototype). Three participants stated that it would be good to register their symptoms combined with registering their SABA use. Two participants told the UX/UI designer and researcher that they wanted to fill out the questionnaire about symptoms multiple times a week and receive a reminder (a) daily or (b) three times a week. The CARAT [48] is used to register symptoms in the app, which measures symptoms weekly. Therefore, the development team implemented a weekly reminder to fill in the questionnaire in the app’s first version.

All participants mentioned that they wanted to look up information about inhalation use, medication, and/or how to properly take their medication (i.e., correct use of their inhaler). Given that the correct use of inhalers and medication adherence is essential for asthma control, the development team decided to add information about medication and inhalation use. One participant also mentioned that it would be helpful to have a description of the app and its functionalities. This was also added by the development team.

All participants received the question of whether they would use the app. All participants stated that the app would only match their needs when they could register all asthma medication; however, due to the project’s scope, we could not implement this remark. Nevertheless, 75% of the participants declared that the app would be helpful to take to their general practitioner or pulmonologist to show the results in the graph. Participants also indicated that it would take too much time to keep track of their medication without an app.

Participants also got a question about what they missed in the app or would like to change. They all wanted to fill in details, such as having the flu, as a note. Three participants mentioned a counter would be helpful (i.e., to count the number of inhalations left in the inhaler); however, the development team decided not to add a counter, because different inhalers have different dosages and most patients have multiple inhalers in different places (e.g., at home, at work, in the gym bag). Therefore, a counter would not be practical to implement in an app.

#### Pharos Session

The two low-literate asthma patients mentioned similar remarks as the other participants from the qualitative test session. It was unclear for the two low-literate asthma patients that the icon on the top left of the landing page would navigate to the questionnaires, the term SABA was unclear, and in the graphical overview, it was unclear whether a score was good or bad, and what the score of the questionnaire exactly meant.

The participants with low literacy also brought up new points. They did not understand the link between the background visual of the landing page and asthma or medication. They also wanted a further explanation of the medication and the risks of taking SABA more than twice a week and an explanation of what they should and should not do in terms of their medication. More information about medication and medication use could clarify what a user should do in certain situations, and this would also create peace of mind among worried users. Finally, the term “symptoms” was used during the session instead of “triggers”. The participants mentioned that the term “symptoms” in the context of triggers (e.g., dust and animals) was unclear.

### 3.2. Second Iterative Cycle

The first impressions of the app were identified. Two participants mentioned that it was great that they could change their maximum amount of SABA, based on their doctor’s advice instead of only following the existing guidelines. One of these participants also stated that the hot air balloon was a beneficial change. The participants at this stage already mentioned a few improvements; for example, the CARAT score was not completely clear after filling in the questionnaire. The development team decided that additional written information (on communication level 1B on the scales of the Common European Framework of Reference for Languages [49]) should be implemented to clarify the score. All remarks about the first version of the app can be found in Table 2. The non-applicable remarks can be found in Appendix A, Table A2.

Overall, participants were positive about the homepage (e.g., it is clear how much SABA you have used, and you can easily add a SABA dose) and the graphical overview. However, a few negative aspects were mentioned during the interviews. The term SABA could be unclear for users, and the baseline question about the maximum amount of SABA was ambiguous. These remarks were addressed. Furthermore, one participant misinterpreted the CARAT score in the graphical overview. The development team implemented extra information to aid the score interpretation in the graphical overview. This information was also added in the information section on the subpage, where the app was further explained. Participants were asked about any errors they encountered; no errors came across.

The participants were asked about the ease of use of the functionalities. All participants mentioned that all four icons and the matching functionalities were easy to find and easy to use. Moreover, the participants mentioned that the different functionalities, such as the graphical overview and the information section, were suitable. One participant stated that she wanted to know that she should fill out the CARAT. The development team added a pop-up with a button to the CARAT on the homepage after registering the first SABA use of the week.

Participants received the question about what they believed the app’s aim was. Most participants mentioned that the apps’ aim was to help users get insight into SABA use, not use too much SABA, and get insight into the stability of asthma. One participant mentioned that the aim should become clear when first using the app. The development team implemented this at the start of the app, shown to users who open the app after installation.

All participants were asked about text comprehensibility. All participants mentioned that they thought the texts were comprehensible. One participant suggested adding links to the sources in the information section, and the development team added these links.

As in the first iterative cycle, participants received the question about missing aspects of the app. Similar to the first iterative cycle, most participants (60%) mentioned that they wanted to add other or all medicines. One participant stated that additional medication could also be written down by the app user in the note section, and this suggestion was added to the app. One participant mentioned that she wanted to revisit the filled-in CARAT. It was not possible to address this remark, but the subscores (i.e., score of the upper airway and score of the lower airway) were added to the total score to provide more information. Users were also able to find an explanation of the scores in the graphical overview by pressing the information icon.

The majority of the participants (60%) would use the app when released because the app is user-friendly, and the overview of used SABA is convenient. Moreover, the app provides insight into the fluctuating periods of asthma (i.e., the overview shows when you had a worse period). It is also helpful to show the app to the pulmonary nurse. Two participants stated that they would not use the app at the moment because it had no added value. One participant would use the app when her pulmonary consultant would have access to the app. The other participant mentioned that she would not use the app because not all medication could be registered. Both remarks were not implemented as the remarks felt outside the project’s scope.

## 4. Discussion

The purpose of the current study was to describe the development of an application that promotes responsible SABA use in people with asthma, using a participatory design. Compared to traditional development processes [40], such as state-based models and agile methods, the participatory design process includes user input throughout the development stages instead of only including requirement activities, design, implementation, testing, deployment, and maintenance. In this study, two iterative cycles were incorporated into the development process, and different stakeholders provided input in the development phases. The majority of the participants found the app helpful. The app’s final version allows end-users to register SABA use and track their asthma symptoms, including related triggers. A graphical overview shows how these factors are associated with one another. Moreover, psychoeducation is offered, covering different asthma-related topics. Medication use is compared to the medical guidelines or, when applicable, to the advice given by the users’ healthcare professionals. This study showed that engaging end-users in app development is essential. The participants contributed valuable information that was crucial for the app development. Problems frequently encountered by app users, such as the inability to find the information they were looking for and insufficient information [39,52], were indeed mentioned by the participants during the development process. It was possible to adapt the app to match the users’ needs by addressing the end-users’ feedback during development.

The developed app can help patients gain more insight into their SABA use, which is essential to addressing the problem of SABA overuse [5,6,7,21]. Furthermore, the app provides insight into the relationship between asthma symptoms and exposure to personal asthma triggers by combining these factors in a graph. It can help patients identify what triggers are (often) associated with a symptom increase and might need to be avoided (if possible). Studies have shown that personalized self-management apps may support patients in dealing with their disease daily [24,25]. An app such as the Asthma app could help to reduce the frequency of SABA use, increase SABA-free days, and improve asthma control [23,24]. Furthermore, end-users can learn to recognize triggers and discuss the relationship between the triggers, symptoms, and SABA use with their physician. One recommended step to prevent SABA overuse is appropriate patient education, especially for patients who did not receive correct advice about SABA [53]. The Asthma app may help the physician to get more insight into the SABA use of their patients and could be used to create an asthma self-management plan and align treatment. Due to the involvement of different stakeholders during the development process, the development team tried to ensure user engagement and reduce SABA overuse over time.

A limitation is that a selective (i.e., only female) small group of participants participated. It is possible that one or both of the low-literate asthma patients were male; however, this information was not shared with the researchers. The app might thus not fit the needs of all patients (e.g., males with asthma). As the burden of disease is higher in older male adults (≥50 years old) than in females in the same age group [1], it is crucial to identify whether the app is also suitable for males. Although the current study is based on a small sample of participants, Barnum [54] concluded that a minimum of five participants is sufficient when there is close collaboration and mutual understanding between the research and development team. This was the case in the current study. To get a better insight into the app’s usability and effectiveness, we are currently conducting a pre-post test study. All participants who download the app are invited to join the study. This study aims to better understand the app’s usability and effectiveness in a larger sample.

A second limitation is that not all end-users were involved in all phases of the development; that is, only three participants were involved in the qualitative test session and the interview. Therefore, we were unable to check whether the feedback from these participants was adequately addressed. Nevertheless, the individuals who only participated in the second iterative cycle gave valuable insights that may not have been obtained when only using the participants that were part of the first iterative cycle. Low-literate patients were not involved during the second iterative cycle. As a consequence, the information section was not tested by this group. Nevertheless, the app’s content was developed with low-literate individuals in mind (i.e., information was written on communication level 1B on the scales of the Common European Framework of Reference for Languages [49]).

A third limitation of this study is that the development team could only create a minimal viable product. Some features requested by participants, such as registering all medication, fell outside this project’s scope and were therefore not implemented in the current version of the app. Even though not all user requirements could be incorporated into the app, it helps to identify what is important for end-users and how the app can be improved in the future. Moreover, the participants believed that the app would be helpful for them. It is important to realize that an app is not a static product but needs to be regularly evaluated and adjusted where necessary. Therefore, requested features may be implemented at a later point in time. A development team should be aware that an app’s continuous development and maintenance are associated with ongoing costs. Especially when there is no or limited funding, costs are considered a barrier when implementing an app [52,55]. The amount of funding that is needed for app development and maintenance will vary between projects because of, amongst other things, the complexity of an app. For example, an app with only written information is easier to develop and maintain than an app where patients can contact their HCP or monitor health data. Therefore, it is essential to consider the maintenance costs before starting the app development process.

## 5. Conclusions

An application was developed that promotes responsible SABA use in people with asthma using a participatory design. It was considered helpful by end-users who co-created the app. Feasible remarks made by end-users, such as the possibility to adjust the maximum amount of SABA, were implemented in the app’s final version. Further research is being carried out to establish the app’s usability and effectiveness; the app’s preliminary effect on improving weekly SABA use at three months compared to baseline will be examined.

## Figures and Tables

**Table 1:** Overview of remarks about the app prototype and the frequency and feasibility of these remarks.

Remarks Prototype	Frequency	Feasibility ^1^
**Registering SABA (task: add one SABA inhalation)**		
The amount of SABA advised is personal (“I need to use SABA more than once a day already”)	2	+
The icon on the top left seems to be for a calendar	1	+
Is the data automatically sent to the physician?	1	−
**Balloon**		
Bursting balloon does not deter	3	+/−
I am feeling punished when I am allowed to only use SABA twice per week, as I already need two inhalations in the morning	1	+
The balloon is clear, but the bursting is stigmatizing	1	+/−
A balloon that is getting bigger is not equal to getting less air. I also have a negative association with the trees in the background because of hay fever	1	+/−
**Graph**		
Possibility to view the data (graph) per 6 months	3	−
Possibility to view the data (graph) per month	2	+
The graph is not clear, what is the Y-axis? Lower seems to be worse, but it is not clear	2	+/−
Possibility to view the data (graph) per season	1	−
**Questionnaire (not available in app prototype)**		
I want to receive a notification to fill in the questionnaire at the start of the week, and receive a reminder on Wednesday and Friday	1	−
I want to receive a reminder daily because after two days I do not know anymore that I need to fill out a questionnaire	1	−
Can you see the items that you have filled in? So actually being able to view the questionnaire each time you completed it (for example, which answer did I give on having a cold that week)	1	−
I expect a different questionnaire per asthma type	1	−
Missing to fill in pain	1	−
**Information (not available in the app prototype)**		
About inhalation use or medication (explaining short-and long-acting respiratory inhaling)	4	+
How do you properly take your medication	3	+
News items or YouTube videos	2	−
Adding information from a specific website about prescribing and pharmacology	1	−
Extra information about the app/how does the app work	1	+
**Answers “would you use the app?”**		
Only relevant when I can register all asthma medication	4	−
**Missing aspects or wanted changes in the app**		
Add a note to fill in details, such as having the flu	4	+
The maximum amount of SABA should be adjustable per user	3	+
A counter is helpful	3	−
What if you use a combination of different medicines?	3	−
Matching prescription and maximum dosage in-app	2	+
Adding the word “week” in the graph, what does 1/2/3 et cetra mean?	2	+
I want to know all the side effects of each type of medication	2	−
Different types of asthma are important	2	+
A lower score in the graph in combination with a higher SABA use does not seem logical	1	+/−
Adding details per day	1	−
Adding change of medication and taking notes of different side effects, which can be forwarded to the physician	1	−
Receive a reminder at the end of the day and being able to notify the app about the severity of the asthma	1	−
Keeping the notification casual	1	+

^1^ The remarks have been split into three categories: feasible (+), feasible, but needed to be modified (+/−), and not feasible (−); it would not be implemented, because (a) it would take too much time to develop or (b) not fit the scope.

**Table 2:** Overview of remarks about the first version of the app and the frequency and feasibility of these remarks.

Remarks First Version of the App	Frequency	Feasibility ^1^
**First impression**		
I want to fill in the CARAT more often than once a week	2	−
The app is very much focused on SABA use, I would prefer to add all asthma medication	2	−
The score of the CARAT was not completely clear (“When filling out the questionnaire, there was a bit of a confusion whether the red score was okay or not”)	1	+/−
I wanted to change notes from the first week in the second week, but I was unable to do this. I would have preferred to add a few things, such as my other medication use	1	+/−
**Evaluation app usage**		
I do not think I would use the app in my daily life. I am alert when I need to take more medication and what the causes are of the extra usage. If I need to take more medication over a longer period of time, I contact my lung consultant. I do not need to put this information in an app. I would only use the app if my lung consultant could also have access to my data	1	−
I do not know whether SABA is a commonly known term	1	+
You do not receive an alert when you have taken too much SABA or when you have uncontrolled asthma	1	−
Maybe add extra information at the CARAT: that these scores need to be taken seriously and that it is your own responsibility	1	−
It is nice to notice that all my CARAT scores are below the dotted line	1	+
I needed to get used to the hot air balloon. The add-sign was not immediately clear, I noticed this button on the second day of usage. Maybe you can add a manual at the start of the app? Especially for users who are less technical	1	+/−
The first question about your SABA intake was unclear. I filled in the amount of SABA I can use per day instead of per week (4 inhalations versus 28 inhalations)	1	+/−
**Ease of use functionalities**		
I found out on the second day that I needed to fill out a questionnaire about symptoms	1	+
There was not a lot of new information in the information section. Maybe add information about the goal of the app?	1	+
At the start the hot air balloon was not clear, but this is negligible	1	−
The add-sign is not very clear	1	−
**Aim**		
The goal is to not to use too much SABA and otherwise contact your doctor. However, what is the next step when you have used your maximum amount of SABA? So is the app purely for tracking medication use? Is it part of your asthma plan? When should you exactly alarm your doctor? It might be useful to add this information in the app	1	−
Yes, it is about SABA. However, it would be convenient to know what the goal of the app is before using it. I needed to go through the whole app before finding the goal	1	+
**Missing aspects or wanted changes in the app**		
I would want to add more/all medicines next to SABA	3	−
I would want to revisit the filled-out CARAT, this way I can discuss this with my pulmonary doctor	1	+/−
Add information about SABA, preferably at the start of the app	1	+
Add or change information in the notes from the previous week	1	−
Is it possible to send the data to my doctor or nurse, this way they can check the graphical overview during an e-consult	1	−
I would add a remark at the notes that users can write down their other medicines over there	1	+
**Using the app after release**		
No, I would not use the app. It has no added value until my long consultant has access to my data	1	−
No, because I want to be able to add all my asthma medication	1	−

^1^ The remarks have been split into three categories: feasible (+), feasible, but needed to be modified (+/−), and not feasible (−); it would not be implemented, because (a) it would take too much time to develop or (b) not fit the scope.

## Data Availability

Data is contained within the article.

## Appendix A

**Table A1:** Overview of non-applicable remarks about the app prototype and the frequency of these remarks.

Remarks Prototype	Frequency	Category ^1^
**Registering SABA (task: add one SABA inhalation)**		
I would use the app immediately after using SABA; otherwise, I would forget to register the used SABA	3	1,2
The homepage is accessible	1	1,3
I always play with new apps first to see how they work	1	2
It is great that the date is prominent on the homepage	1	1,3
**Balloon**		
Warning that you have taken too much SABA is appropriate	3	1,3
Bursting of the balloon looks like a little sun, later it became clear that it means that the balloon has burst	1	1,3
It is a nice design, the app can be used anywhere because others do not immediately see that the app is about asthma	1	1
It seems like an app for children (a lot of gamification)	1	4 *
The use of a traffic light would also be pleasant; if you have taken less SABA than twice a week, the color could be green	1	4 **
**Graph**		
Triggers are clear in the overview	4	1,3
Swiping from the sixth to the second week felt natural	1	1,3
**Questionnaire (not available in app prototype)**		
Registering symptoms in combination with registering SABA use is a good idea	3	1
Pulling apart fatigue/restriction	1	2 ***
**Answers “would you use the app?”**		
Yes, I would use the app, because it takes too much time to keep track of my medication at the moment	3	1,2
Yes, it is useful to take the app to my general practitioner/pulmonologist to show the results in the graph	3	1,2
I would definitely use the app! At the end of the week, you have your results. Where can I find the peak flow? It would help me to change my behavior	1	1,2 ***
Yes, this would increase my energy distribution and I would love to do this without taking medication	1	1,2
I do not need any insight into my medication, because I am a nurse. I would rather know what I need or when I should take more medication	1	3 ****
I can look up my symptoms and share this insight with my physiotherapist	1	1,2
**Missing aspects or wanted changes in the app**		
Make the date more prominent	1	3
Being able to add SABA daily	1	3
Disseminate the app via pulmonary nurses and perhaps engage them in testing the app	1	2 ***

^1^ The non-applicable remarks have been split into four categories: (1) positive remark and therefore no app change was needed, (2) (general) remark that is unrelated to the development of the app, (3) already being incorporated in the app, or (4) other, * Gamification was not included in the app’s prototype, ** The use of the traffic light was already incorporated in a different form (the graph and scores which show (un)controlled asthma) into the app, *** The research nurse (author CH) represented the HCP perspective and deemed, in consultation with the other development team members, the remarks as non-applicable, **** Most users will not have a medical background. Furthermore, information about medication would already have been added by the development team.

**Table A2:** Overview of non-applicable remarks about the first version of the app and the frequency of these remarks.

Remarks First Version of the App	Frequency	Category ^1^
**First impression**		
It is great that you can change the maximum amount of SABA based on the advice of your doctor	2	1,3
The hot air balloon is a beneficial change compared to the bursting balloon	1	1,3
It is a nice and clear app. The app can also be used by less literate people. It is obvious how to use the app	1	1
Great that I can add triggers, this way I get an overview when symptoms worsen	1	1,3
**Evaluation app usage**		
When you open the app, it is immediately clear how much SABA you have used and you can easily add SABA	2	1,3
The information used in the app was clear, such as the different asthma types	2	1,3
It is useful that you can fill in the CARAT questionnaire once a week. The questions are very clear	1	1,3
It is clear that you can change the maximum amount of SABA use under “profile”	1	1,3
The feedback “Great, you needed less SABA than recommended” is pleasant, because it sounds positive	1	1,3
The link to the website www.inhalatorgebruik.nl is very helpful	1	1,3
This app came into my life at the right time and place, due to COVID-19 I am unable to go to my pulmonary rehabilitation	1	1,2
It is very pleasant that the app is solely focused on asthma and that there are no advertisements in the app	1	1,3
The app does not contain too much information, if you want to know more, you should contact your pulmonary nurse or doctor	1	1,2
The dashboard is really convenient. In the graph you get an overview of your symptoms. It is a lot harder to get this overview by writing it down in a notebook, because you sometimes forget to write it down. For example, this week I do not remember how I have slept last week	1	1,3
**Ease of use functionalities**		
All four icons and the matching functionalities were easy to find and easy to use	5	1,3
The graphical overview was easy to interpret	5	1,3
The questionnaire about symptoms and triggers was clear and quickly to fill out	1	1,3
The weekly and monthly graphical overview were both clear	1	1,3
Notes work well as some sort of weekly report	1	1,3
The information section was clear	1	1,3
**Goal**		
Yes, it is clear which information you want from me, namely is my asthma controlled/how stable am I at the moment?	1	1
Yes, I think it is clear	1	1
The goal is clear: how to use your SABA and how you can decrease your SABA use	1	1,3
**Using the app after release**		
Yes, because it shows you an overview of your used SABA and the app is user-friendly	2	1,3
Yes, because it becomes clear when my asthma is fluctuating and when I have a worsen period. Moreover, I can show the app to my pulmonary nurse	1	1,2

^1^ The non-applicable remarks have been split into four categories: (1) positive remark and therefore no app change was needed, (2) (general) remark that is unrelated to the development of the app, (3) already being incorporated in the app, or (4) other.
